# Multilevel Selection in Models of Prebiotic Evolution II: A Direct Comparison of Compartmentalization and Spatial Self-Organization

**DOI:** 10.1371/journal.pcbi.1000542

**Published:** 2009-10-16

**Authors:** Nobuto Takeuchi, Paulien Hogeweg

**Affiliations:** Theoretical Biology and Bioinformatics Group, Utrecht University, Utrecht, The Netherlands; Washington University School of Medicine, United States of America

## Abstract

Multilevel selection has been indicated as an essential factor for the evolution of complexity in interacting RNA-like replicator systems. There are two types of multilevel selection mechanisms: implicit and explicit. For implicit multilevel selection, spatial self-organization of replicator populations has been suggested, which leads to higher level selection among emergent mesoscopic spatial patterns (traveling waves). For explicit multilevel selection, compartmentalization of replicators by vesicles has been suggested, which leads to higher level evolutionary dynamics among explicitly imposed mesoscopic entities (protocells). Historically, these mechanisms have been given separate consideration for the interests on its own. Here, we make a direct comparison between spatial self-organization and compartmentalization in simulated RNA-like replicator systems. Firstly, we show that both mechanisms achieve the macroscopic stability of a replicator system through the evolutionary dynamics on mesoscopic entities that counteract that of microscopic entities. Secondly, we show that a striking difference exists between the two mechanisms regarding their possible influence on the long-term evolutionary dynamics, which happens under an emergent trade-off situation arising from the multilevel selection. The difference is explained in terms of the difference in the stability between self-organized mesoscopic entities and externally imposed mesoscopic entities. Thirdly, we show that a sharp transition happens in the long-term evolutionary dynamics of the compartmentalized system as a function of replicator mutation rate. Fourthly, the results imply that spatial self-organization can allow the evolution of stable folding in parasitic replicators without any specific functionality in the folding itself. Finally, the results are discussed in relation to the experimental synthesis of chemical Darwinian systems and to the multilevel selection theory of evolutionary biology in general. To conclude, novel evolutionary directions can emerge through interactions between the evolutionary dynamics on multiple levels of organization. Different multilevel selection mechanisms can produce a difference in the long-term evolutionary trend of identical microscopic entities.

## Author Summary

The origin of life has ever been attracting scientific inquiries. The RNA world hypothesis suggests that, before the evolution of DNA and protein, primordial life was based on RNA-like molecules both for information storage and chemical catalysis. In the simplest form, an RNA world consists of RNA molecules that can catalyze the replication of their own copies. Thus, an interesting question is whether a system of RNA-like replicators can increase its complexity through Darwinian evolution and approach the modern form of life. It is, however, known that simple natural selection acting on individual replicators is insufficient to account for the evolution of complexity due to the evolution of parasite-like templates. Two solutions have been suggested: compartmentalization of replicators by membranes (i.e., protocells) and spatial self-organization of a replicator population. Here, we make a direct comparison of the two suggestions by computer simulations. Our results show that the two suggestions can lead to unanticipated and contrasting consequences in the long-term evolution of replicating molecules. The results also imply a novel advantage in the spatial self-organization for the evolution of complexity in RNA-like replicator systems.

## Introduction

Consideration of selection acting on multiple levels of biotic organization is important for understanding of biological evolution in general [1]–[6]. In the studies of prebiotic evolution, it has been shown that some form of multilevel selection is even necessary for the maintenance and evolution of complexity in the interacting RNA replicator system [7]–[12].

The RNA-like replicator system is considered one of the simplest chemical systems that can undergo Darwinian evolution in a self-sustained manner [13],[14]. Hence, RNA replicators have been suggested as the central player of prebiotic evolution in the RNA world hypothesis [15]–[18]. Besides whether or not such replicators—or its analogues [19]—can actually exist (see [20]–[25], for recent progress on this), an interesting question is whether such a chemical replicator system can increase its complexity through evolution and approach the biotic system as we know it.

The importance of multilevel selection in prebiotic evolution is based on two problems that arise in the evolution of replicator systems. Firstly, there is a fundamental problem about the accumulation of information in exponentially growing replicators. That is, the maximal length of sequence patterns that can be maintained under the mutation-selection process in a single replicator quasi-species is severely limited by high mutation rates, which are supposed in primordial replication processes based on RNA molecules [13],[26] (see also Text S1 note 1). Hence, a solution to information accumulation has been sought in the symbiosis of multiple species of replicators. As such a solution, a classical study suggested the so-called hypercycle, in which every replicator species catalyzes replication of another species, forming a cyclic network of cooperative interactions [27]. However, there is an inherent problem in such a cooperatively interacting replicator system, in that selection acting on the level of individual replicators favors only the evolution of better templates, but does not favor—even disfavor [28]—the evolution of better catalysts [7],[8]. To overcome this problem, spatial structure in a population has been suggested.

Generally speaking, spatial population structure can be classified according to whether it is implicit or explicit: Implicit population structure arises from the birth-death-migration process of individuals themselves, whereas explicit population structure is imposed to a population by some external boundaries (of course, the external factors can depend on the activity of individuals). In the context of prebiotic evolution, both kinds of population structure have been investigated: (i) spatial self-organization of populations in a diffusion-limited, surface-bound replicator system as implicit structure [4]; (ii) compartmentalization of replicators by vesicles as explicit structure [9],[29]. Historically, the two kinds of population structure were given separate consideration for the interests on its own. In both cases, however, the essential process that makes the difference from a well-mixed system is the formation of multilevel selection, i.e. selection operating on the level of microscopic entities (i.e. individual replicators) and on the (various) levels of mesoscopic entities, such as (spiral) waves and protocells, that arise from spatial population structuring.

In a previous study, we constructed a computational model that can simulate a surface-bound replicator system and compartmentalized replicator system in a unified framework. We therewith investigated the two different types of spatial population structure—explicit versus implicit—with respect to their influence on the macroscopic stability of different evolving replicator systems [30]. The current study aims to extend the previous study: It makes a direct comparison between the spatial self-organization and compartmentalization with respect to their effects on the eco-evolutionary dynamics of a simple interacting replicator system, particularly, by focusing on the interactions between the dynamics of microscopic and mesoscopic entities. For this sake, we adopt the following research strategies. Firstly, we consider an identical model of interacting replicators in the surface-bound system and in the compartmentalized system so that, while the two systems differ in mesoscopic entities, they share identical microscopic entities. Secondly, we design vesicle-level processes such that they do not introduce an extra burden for survival that is independent of the replicator dynamics itself; e.g., we neglect the problem of substrate uptake through membranes (see [31],[32] for experimental studies on this issue). These considerations allow us to focus on the effect of spatial population structure itself. Thereby we study how different types of spatial population structure achieve the macroscopic stability of a replicator system, and how the dynamics of different mesoscopic entities influence the evolutionary dynamics of microscopic entities and vice versa.

## Models

Our model of a compartmentalized replicator system consists of two planes of stochastic cellular automata (CA), where one simulates replicator-level processes, and the other simulates vesicle-level processes. The compartmentalized replicator model (compartment model in short) can be converted to the surface-bound replicator model (surface model) simply by removing the vesicle plane. (See Text S1 for more details on the models.)

### Replicator Model

The replicator model investigated here consists of two types of molecules: replicase and parasite. The replicase can catalyze replication of other molecules, whereas the parasite cannot. The parasite switches between two conformations, viz. folded state and template state. When a parasite is in the folded state, it can facilitate the growth of the vesicle in which it resides (explained later), but cannot be replicated by the replicase; when in the template state, it can be replicated, but cannot facilitate the vesicle growth. We assume the conformation switching is so fast that it is always in equilibrium (see also Text S1 note 2). Hence, the concentration of parasites in the folded state (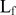) and that in the template state (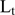) can be expressed as 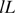 and 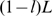 respectively, where 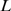 is the total concentration, and 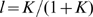, and 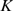 is the equilibrium constant of 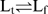. Thus, the current replicator system can be represented as follows:
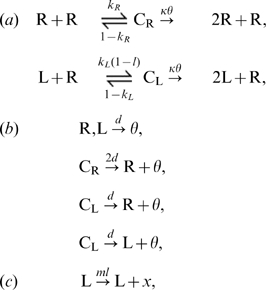
(1)where 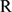 and 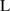 denote a replicase and parasite molecule respectively; 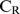 and 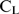 denotes a complex molecule between 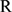 and 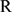 and that between 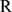 and 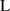 respectively; 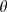 represents the generalized resource for replication. In Reaction 1:

- is complex association/dissociation and replication. We assume that the sum of the rate constants of association/dissociation is fixed and, without loss of generality, set it to 1. 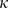 is the rate constant of replication; thus, 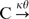 actually means 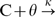. 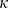 is set to 1 throughout this study, where we assume that the discrimination of templates by replicase lies in the association/dissociation and moreover, that replication and association/dissociation occur at a similar speed.
- is decay, and it is assumed that its rate is invariant. Each molecule forming a complex also decays independently, which can be considered the decay of 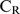 and 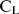.
- is the reaction that facilitates the vesicle growth as explained in Section “Vesicle Model”.

The consideration of complex formation is to take into account the fact that replication takes finite amount of time, during which the replicase cannot be replicated. Complex formation thus considerably disadvantages the replicase over the parasite (see [28] for more details; see also [33]).

The replicator dynamics was modeled in the framework of stochastic cellular automata (CA). The model consists of a two-dimensional grid of 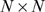 squares, where one square can contain at most one molecule. Empty squares are considered to represent the generalized resource (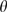), which limits the maximum number of molecules the system can sustain globally and locally. The model's dynamics are run by randomly choosing one grid square and then locally applying the algorithm that simulates Reaction 1 and diffusion (both prohibited to occur across grid boundaries). The reaction and diffusion algorithm employed here simulates the chemical reaction dynamics in such a way that the result approaches that of the Gillespie algorithm in the limit of 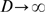 with 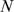 being constant, where 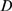 denotes the rate of diffusion.

To investigate the evolution of replicators, we introduced “mutations” in 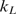 and 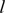. A newly produced parasite inherits the values of 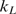 and 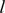 from its template, but mutation can modify either 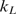 or 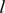 by adding to it a number uniformly distributed in 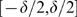. Moreover, 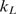 and 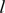 are bounded in 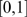 (see also Text S1 note 3). The mutation of 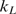 and 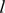 occur with a probability 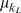 and 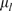 respectively, and they are mutually exclusive.

### Vesicle Model

Vesicle-level processes were modeled by using the so-called Cellular Potts Model (CPM) [34],[35]. The CPM is a two-scale stochastic CA: It explicitly defines a mesoscopic entity, “vesicle”, by a set of grid squares having an identical state; at microscopic scale, the updating rules tend to minimize the interface between different vesicles, while at mesoscopic scale the updating rules tend to keep the volume of a vesicle (i.e. the number of the squares constituting a vesicle) at the target volume.

We implemented a two-dimensional CPM of 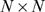 squares. We then superimposed it onto the replicator CA plane and coupled the dynamics of the two as follows. Firstly, the molecules in the replicator CA are forbidden to permeate though the vesicle boundaries in the CPM (i.e., replicators cannot diffuse across vesicle boundaries, and vesicle boundaries cannot move over molecules). Secondly, the dynamics of the target volume of vesicles are governed by the increase due to the occurrence of Reaction 1(c) and the decrease due to spontaneous decay. That is, if Reaction 1(c) happens inside a vesicle, its target volume is increased by 1 (if it happens outside vesicles, it is ignored); hence, Reaction 1(c) can be considered to represent membrane production. Moreover, the target volume 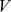 decays with rate 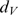 (the decay rate of target volume). It is worth noting that 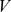 plays a crucial role for the competition among vesicles because the greater 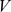 is, the greater chance a vesicle has to expand its actual volume and grow. Thirdly, a vesicle divides when its actual volume exceeds a threshold 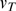 (“reproduction”): A vesicle is divided along the line of the second principle component; the internal replicators are distributed (or “inherited”) between the two daughter vesicles according to the location (if a complex is divided between the two vesicles, it is dissociated); and the target volume is also distributed between them in proportion to their volume after division. It is also worth noting that 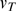 determines the size of vesicles and, thus, the number of replicators in vesicles, which is a crucial parameter of compartmentalization. Additionally, the death of vesicles is not simulated as an explicit process, for it happens implicitly through the dynamics already specified (see also Discussion).

Importantly, our compartmentalized replicator model ignores the transport process across vesicle boundaries and the resource for the target volume growth. This simplification is to avoid introducing an extra constraint for survival that is not considered by the replicator model per se (cf. the surface model), which allows us to directly compare spatial self-organization and compartmentalization with respect to their effects on the replicator dynamics. Also notable is that vesicle-level selection is nearly at optimal efficiency, for a difference in 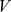, which is unbounded, is always reflected in the competence in volume expansion.

## Results

This section is organized in seven parts. Firstly, we explain that the replicator system without spatial population structure is evolutionarily unstable. Secondly, we show that the two models with different spatial population structure—i.e. the surface model and compartment model—allow the evolutionary stability of the replicator system. Moreover, they display an emergent long-term evolutionary trend which is inconceivable in a well-mixed system. To understand these results, in the third and forth section, we analyze each model separately. In the fifth section, we compare the findings from the two models and delineate the similarities and differences between them. In the sixth section, we turn our attention to the condition under which the two models display the macroscopic stability.

### Evolutionary Dynamics of Replicators without Spatial Population Structure

The dynamics of the replicator system without population sturcutre can be considered the point of reference for the dynamics with population structure. A simple ordinary differential equation (ODE) model was constructed that describes the well-mixed system of one replicase and one parasite species according to Reaction 1:
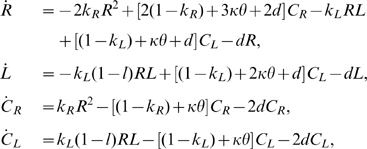
(2)where 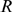, 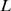, 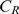 and 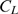 denote the concentration of R, L, 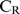 and 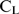 respectively; and the dots denote time derivative; and 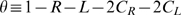.

**Figure 1:**
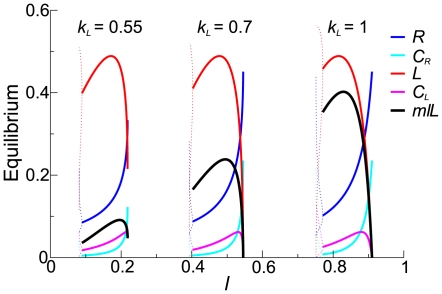
Bifurcation diagram of the ODE model (Eqn. 2). Colored lines represent the stable attractor of each population as a function of 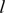 for various values of 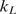: solid lines represent stationary attractors (equilibria), whereas dotted lines represent the maximum and minimum of stable limit cycles (oscillatory solutions), where colors are as designated in the graph. Black lines represent the rate of Reaction 1(c) at equilibrium, i.e. 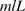 where 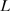 corresponds to the red lines (the values in the region of limit cycles are not shown). The graph omits to display stable equilibria in which only R exists (which occurs for too small 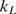 or too great 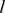) and unstable equilibria. The parameters were as follows: 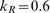; 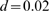; 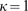, which were used throughout this paper. These parameters mean that one replicase molecule can replicate a template, on average, for 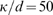 times in its lifetime if finding a template and substrates are extremely fast processes.

We study the behavior of Eqn. 2 as a function of 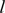 and 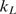 since we later investigate the evolution of these parameters in the systems with spatial population structure. We numerically calculated the equilibrium of Eqn 2 as shown in Fig. 1. The result shows that the values of 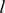 that allow the stable coexistence of 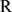 and 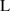 shift to higher values as 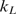 increases. The result can be explained with ease: Increasing 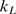 gives an advantage to the parasite for replication; hence, in order to allow the coexistence, this must be compensated by increasing 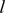, which reduces the fraction of time the parasite spends as template. From this argument, one can also expect that if a parasite species with a greater 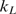 and/or smaller 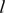 value is introduced to the system, it will out-compete the original parasite; i.e., there is selection pressure to increase 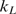 and to decrease 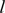. Indeed, this was numerically confirmed by extending Eqn. 2 to include another parasite species (data not shown; the extended version of Eqn. 2 is shown in Text S1). Therefore, if the evolution of 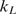 and/or 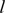 is allowed, a well-mixed system will eventually go extinct due to the evolution of too harmful parasites. This was also confirmed by the CA model simulating a well-mixed system (data not shown).

In summary, the well-mixed replicator system is evolutionarily unstable, so that some sort of spatial population structure is necessary for the feasibility of the evolving interacting replicator system (see [12] on this point discussed with the model explicitly considering the genotype-phenotype-interaction mapping of replicators; see [28] for more detailed analysis on a similar ODE model).

### Evolutionary Dynamics of Replicators with Spatial Population Structure

In this section, we examine the evolutionary dynamics of the replicator system with the two types of spatial population structure, viz. compartmentalization and spatial self-organization. We will examine whether the replicator system can survive despite the evolution of parasites and what kind of evolutionary dynamics the system will display.

The surface model and compartment model were initialized by inoculating the system with small populations of the replicase and parasite of an equal size. 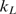 and 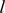 were allowed to mutate (the initial population was homogeneous), while other parameters were fixed. In the compartment model, molecules were randomly placed inside one large vesicle. In the surface model, replicase molecules were placed in a half circle, and parasite molecules were placed in the other half circle. In the compartment model, the value of 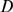 (diffusion) was set so great as to remove the effect of spatial self-organization within vesicles in order to simplify the comparison with the surface model (see also Text S1 note 4); otherwise, the two models had identical values in the shared parameters.

**Figure 2:**
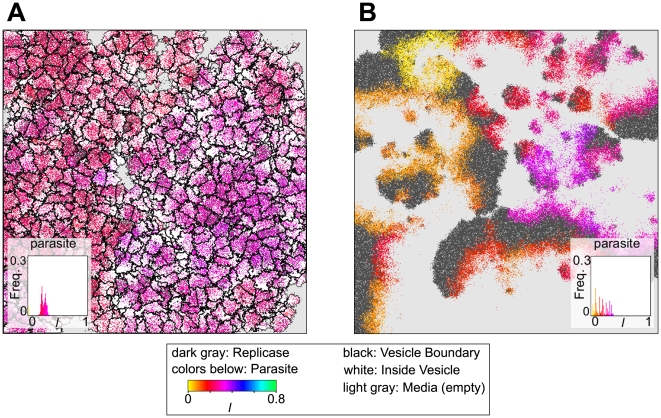
Snapshots of an early phase of simulations. **A:** The compartment model shows the variation in the size and shape of vesicles as well as their replicator content. The histogram depicts the frequency of vesicles as a function of the average 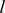 value of the parasites in each vesicle (100 bins). **B:** The surface model. The histogram depicts the frequency of parasites as a function of 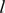 (100 bins). Note that the value of 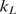 is not depicted in these pictures. The size of the CA was 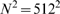 squares in both models. The parameters common to both models were as follows: 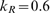; 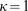; 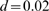; 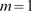; 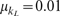 and 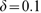; 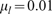; and 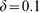. In the surface model, 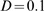. In the compartment models, 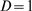, 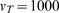 and 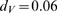.

To obtain the visual image of our models, snapshots of the simulations are shown in Fig. 2. Moreover, Videos S1 and Video S2 depict the spatio-temporal dynamics of the compartment model and that of the surface model respectively (for visibility, Video S1 depicts a smaller scale simulation than that shown in Fig. 2). As Video S2 (surface model) shows, mesoscopic patterns—namely, traveling waves—emerge through the spatial self-organization of the replicator population, which contrasts with the compartment model where mesoscopic patterns—i.e. vesicles—were externally imposed (see [28] for more description on the spatio-temporal dynamics of such waves). In the compartment model, a vesicle expands its volume as the internal replicators multiply, extending into an empty area or pushing other vesicles away (i.e. inter-vesicle competition), and it divides when its volume exceeds the threshold 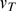. Once a while, a vesicle also shrinks—or gets squeezed—and disappears from the system (i.e. dies) in concurrence of the extinction of the internal replicators.

**Figure 3:**
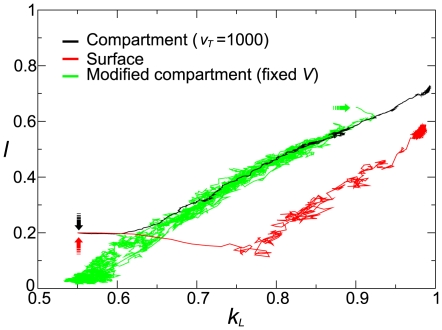
Evolutionary trajectory of 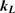 and 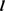. The lines represent the average value of 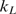 and 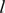. The colors represent the different models as designated in the graph. The arrows point at the initial value of 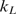 and 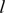. For the compartment models, the average of 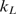 and 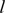 was calculated first for each surviving vesicles (i.e. those contain at least two replicase molecules and one parasite molecule), and these averages were again averaged over all vesicles. The parameters were the same as in Fig. 2.

The long-term behavior of the simulations is depicted as the evolutionary trajectories of the population average of 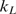 and 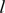 in Fig. 3 (black and red lines). The trajectories can be separated into two phases: short-term evolution and long-term evolution. In the former, the trajectories go to a contour that gives a (mathematical) functional relationship between 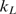 and 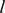, which indicates the emergence of a trade-off situation in parasites regarding the affinity towards the replicase (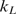) and the availability of templates (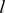). In the latter, the trajectories go along the contour, increasing 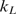 and 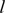. These results show that both the surface model and the compartment model allowed the stable coexistence of the replicase and parasite despite the evolutionary instability of the replicator system explained before. Moreover, the two models exhibited a qualitatively identical evolutionary trend such that the parasite, through evolution, increased the fraction of time it spent in the folded state while it also increased the affinity towards the replicase. This result is surprising, given that the folded state has no predefined functionality in the surface model (in fact, it prevents the replication), whereas it does have a predefined functionality in the compartment model (i.e. to facilitate the vesicle growth).

To understand these results, we next delve into each model.

### Analysis of the Compartment Model

#### Short-term evolution

**Figure 4:**
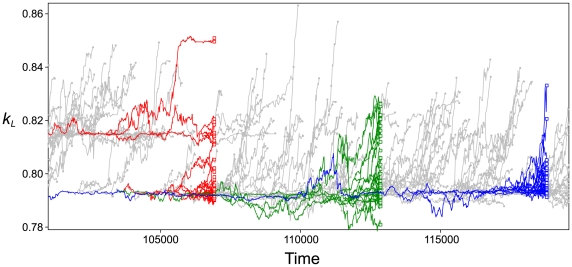
Life history of vesicles. The dynamics of 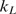 averaged over all parasites in a vesicle were followed for each vesicle in the system. 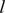 was fixed to 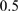. Gray lines indicate 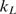 of all vesicle lineages. For visibility, the dynamics of 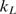 are not shown between the last division and the moment of a vesicle's death. The gray dots indicate this by designating the moment of such last division events. The colored squares indicate all vesicles that were alive at the designated time. The colored lines indicate the ancestral linage of the vesicles indicated by the squares (ancestor trace). Time (abscissa) is scaled to match that of the ODE model with identical rate constants, which is also the case in all figures of this study (precisely speaking, simulation time-steps of the replicator dynamics were divided by 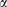, which is defined in Text S1). The size of the CA is 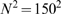 squares. The parameters were the same as in Fig. 2 except 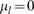 and 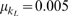 (if 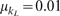 the system died out due to a small number of vesicles). 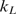 is initially set to 0.8.

To understand how the compartmentalization enables the stable coexistence of the replicase and parasite, we followed the evolutionary dynamics of the internal replicator system of each vesicle. For simplicity, we analyzed a case in which 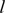 was fixed. As seen from Fig. 4 (gray lines), the evolution of replicators within a vesicle tends to increase 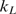. However, the vesicles that have greater 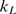 values cannot successfully leave offspring. Therefore, all vesicles existing at some time are the descendants of those that had a small value of 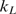 (colored lines).

As we saw previously, selection on the microscopic level (individual replicators) favors the evolution of stronger parasitism (greater 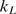 or smaller 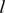). This is also true in the internal replicator system of vesicles. Moreover, a vesicle containing too strong parasites has shorter longevity or lower fecundity (see the next section). Hence, a single vesicle containing replicators inside is an evolutionarily unstable mesoscopic entity. In other words, a vesicle experiences a sort of “aging” (or “maturation” [30]) due to the evolution of the internal replicator system, which has comparable timescale to the lifetime of a vesicle as we will see in the next section. However, the evolutionary dynamics of replicators in a vesicle are highly stochastic due to a small replicator population size and the disturbance from the division of the vesicle [9]. This stochasticity generates variation in the extent of parasitism (i.e. the values of 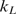 and/or 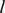) among vesicles, on which selection operates (i.e. vesicle-level selection), disfavoring the evolution of too strong parasitism in a vesicle population. Thereby, the stability of the replicator system as a whole is obtained (i.e. macroscopic stability).

#### Long-term evolution

In the earlier section, we saw that 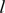 and 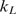 evolved to higher values when both were allowed to mutate. We seek to explain this by the vesicle-level selection. The vesicle-level selection operates on two kinds of variations: variations in the target volume (i.e. the frequency of the occurrence of Reaction 1c) and variations in the stability of the coexistence between the replicase and parasite in the internal replicator system. One the one hand, the former influences the growth of vesicles for the obvious reason and the death of vesicles because a vesicle can die from too small target volume (it can be “squeezed” by nearby vesicles having greater target volume; it can also “shrink” because the growth of its target volume cannot compensate the spontaneous decay). On the other hand, the latter influences the death of vesicles because a vesicle can die from the loss of the coexistence between the replicase and parasite in the internal replicator system, which can be either because of the deterministic instability (Fig. 1) or because of stochasticity due to small population size.

To separate these two kinds of variations (i.e. target volume and the stability of the coexistence), we modified the model such that the target volume is set to a constant value as long as a vesicle contains at least one parasite molecule. This modification deletes the variation related to the target volume, so that the vesicle-level selection now operates solely on the stability of the coexistence between the replicase and parasite in internal replicator systems, which determines the longevity of vesicles. The evolutionary dynamics of the modified compartment model was investigated in the same manner as in the original model. As Fig. 3 (green line) shows, the modified model also allows the stable coexistence of the replicase and the parasite, and its evolutionary trajectory largely overlaps with that of the original model. From this, we can conclude that if the growth of the target volume is sufficiently great (i.e. 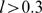 in Fig. 3), the location of the trajectory (i.e. the trade-off between 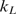 and 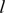) is determined solely by the selection acting on the stability of the internal replicator system.

Interestingly, the result also shows that the modified compartment model displayed an evolutionary trend that is opposite to that of the original model (Fig. 3); namely, the parasite, through evolution, decreased the fraction of time it spent in the folded state (i.e. decreasing 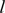) while it decreased the affinity towards the replicase (i.e. decreasing 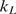). This result indicates that the direction of evolution in the original compartment model was caused by the selection operating on the target volume. Moreover, importantly, the compartmentalization of replicators by itself imposes selection pressure that can act in the opposite direction from that imposed by the predefined functionality in the folded state of parasites to facilitate the vesicle growth. This contrasts with the case of the surface model, where no functionality was predefined in the folded state, but the model nevertheless displayed the same evolutionary trend as the original compartment.

**Figure 5:**
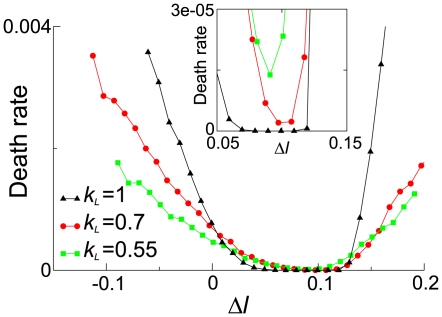
Death rate of vesicles due to the extinction of internal replicators as a function of 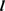 for various values of 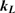. The measurement of the death rate was done through the simulation where the expansion/shrinkage and division of vesicles were forbidden. The volume of vesicles were set to 420, which is approximately the average volume during the evolution simulation of the original vesicle model. A vesicle is considered dead if it contains no molecules or contains only replicase molecules (the frequency of the latter increases as 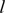 increases). The abscissa 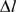 is defined as 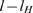, where 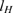 denotes the value of 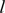 at which the dotted lines and the solid lines meet in Fig. 1 (i.e. the Hopf bifurcation point of Eqn. 2). As seen from Fig. 1, if 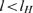, the equilibrium for which the replicase and parasite coexist is locally unstable. If 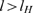, this equilibrium is locally stable. The actual value of 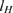 is as follows: 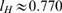 for 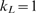; 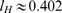 for 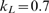; 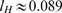 for 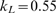. The parameters were the same as in Fig. 1 (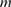 and 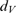 are irrelevant here). The inset is a magnified part of the main graph.

Our next aim is to understand the link between the longevity of vesicles, the stability of the coexistence in internal replicator systems and the values of 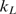 and 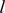. As the first step, we measured the death rate of vesicles as a function of 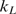 and 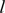. For this sake, we removed the growth and division process of vesicles from the model, while still letting vesicles constrict the diffusion of replicators. We then measured the rate at which vesicles lose the coexistence of replicators in the internal system for various values of 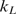 and 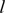. In Fig. 5, the measured death rate is plotted as a function of 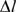, where 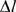 has the following meaning: If 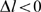, the coexistence of the replicase and parasite is deterministically unstable (Eqn. 2), whereas if 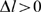 (but 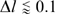), the coexistence is deterministically stable (for more details, see Fig. 5 caption).

**Figure 6:**
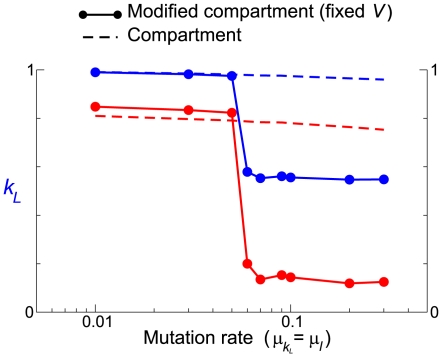
Transition between the survival of the fittest and the survival of the flattest. The figure shows a sharp transition in the long-term evolutionary trend in the modified compartment model, where the target volume is fixed, as a function of the mutation rate (solid lines with filled circles). The survival of the flattest happens for greater mutation rates as explained in the main text, whereas the survival of the fittest happens for smaller mutation rates. Such a transition does not occur in the original compartment model (dashed line) because the selection pressure arising from the functionality of the folded state of the parasite (to increase the target volume) is independent of the mutation rate. For the sake of computational speed, 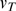 was decreased to 100 in the simulations shown here (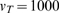 in the preceding simulations), which increased the mutation rate at which the transition happened (because it reduced the effective mutation rate per vesicle). In the modified compartment model, 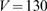 (target volume), 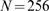 (system size). The other parameters were the same as in Fig. 2.

Interestingly, the result shows an apparent contradiction: the smallest death rate is obtained if 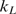 is greater, which cannot explain the evolutionary trend of the modified compartment model. However, a closer look reveails the following: If 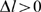 (i.e. the coexistence is deterministically stable), the death rate is smaller for greater 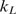. Contrastingly, if 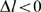 (i.e. the coexistence is deterministically unstable), the death rate is smaller for smaller 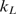. Moreover, as Fig. 4 shows, the timescale of the evolution of the internal replicator system—the maturation of vesicles—is comparable to that of the death of vesicles, which can be interpreted as a high degree of vesicle-level somatic mutation. These results indicate that the vesicle-level selection caused the survival of the “flattest” (i.e. where the death rate increases most moderately as 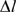 decreases) rather than the fittest (i.e. where the death rate is smallest), which is known to happen if the mutation rate is sufficiently large [26],[36]. Moreover, the above argument implies that when the mutation rate is sufficiently small, the modified compartment model should display the survival of the fittest, which is indeed the case as shown in Fig. 6.

**Figure 7:**
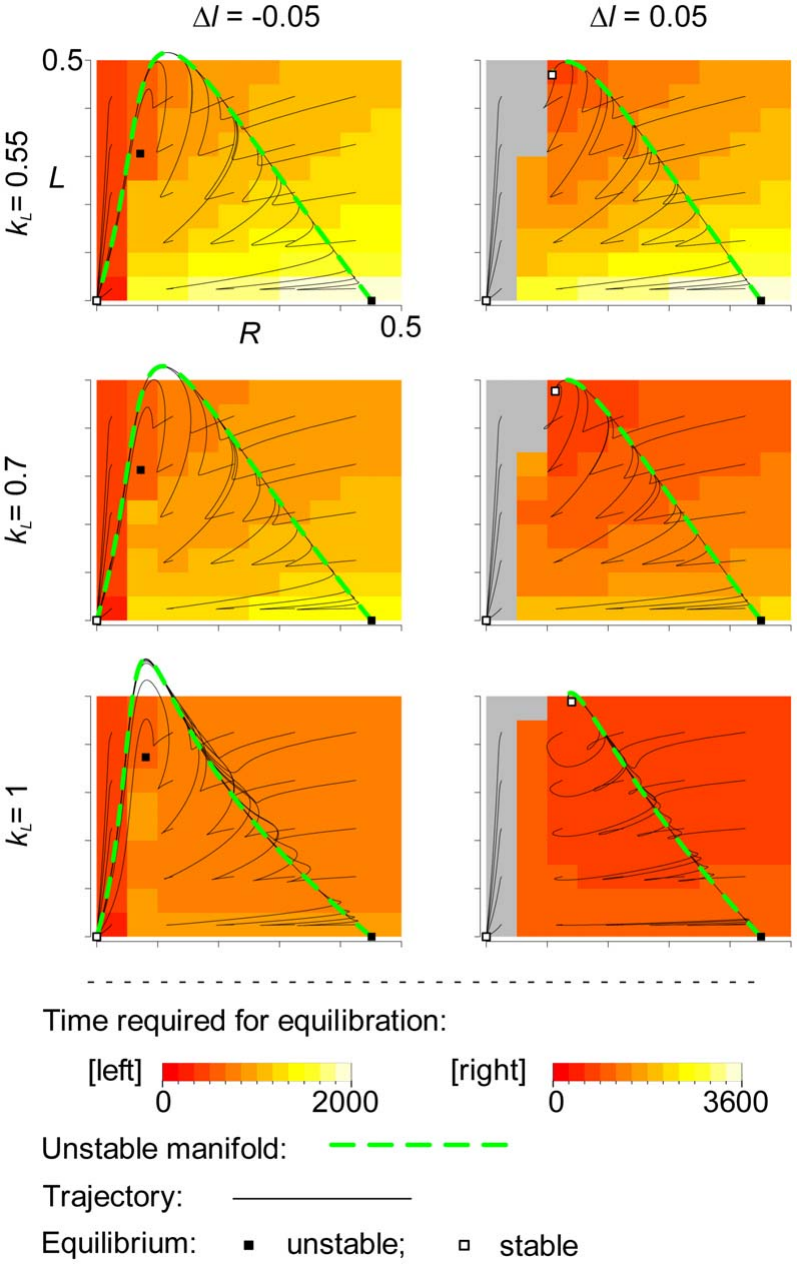
Phase portrait of the ODE model (Eqn. 2) projected to (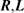)-space for various values of 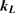 and 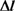. 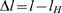
 where 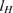 is the Hopf bifurcation point (see Fig. 5). The initial conditions of all trajectories were set to 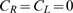, and 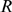 and 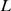 as designated in the graph. The same initial conditions were used to measure the time required for equilibration. The system was considered equilibrated when every variable becomes different from the stable equilibrium by less than 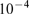. The gray regions indicate that the system went an alternative stable equilibrium (extinction). The parameters not shown in the graph were the same as in Fig. 1.

Having established the phenomenological explanation, we next seek for more mechanistic understanding of the relationship between the vesicle death rate and the values of 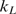 and 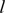 in terms of the stability of the coexistence in internal replicator systems. For this sake, we turn to the property of the dynamical system described by Eqn. 2. An obvious factor that can possibly determine the stability of the replicator coexistence is the local stability of the equilibrium for which the replicase and the parasite coexist. However, this line of analysis turned out to be unsuccessful: the real part of the greatest eigen value of the Jacobian matrix behaves almost identically as a function of 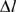 for different values of 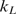 (data not shown; see also Text S1 note 5). This prompted us to look at a more global property of the phase space of Eqn. 2. Fig. 7 shows the time required for equilibration, which represents the speed—and therefore strength—of deterministic flow in the phase space. As seen from the figure, if 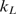 is greater, the deterministic flow is stronger. Therefore, if 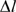 is so large that the replicator coexistence is deterministically stable, increasing 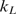 stabilizes the coexistence (and increases the vesicle longevity) because it relatively weakens the effect of stochasticity, which can bring the replicator system to extinction. However, if 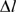 is so small that the replicator coexistence is deterministically unstable, decreasing 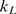 stabilizes the coexistence because it relatively strengthens the effect of stochasticity, which can disturb the deterministic extinction. Previously, we saw from Fig. 4 and 5 that the evolutionary dynamics of internal replicator systems were fast relative to the generation time of vesicles (i.e., the vesicle-level somatic mutation rate was high). In such a case, the internal replicator system is likely to be in the state in which the replicator coexistence is deterministically unstable (i.e. 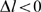); therefore, decreasing 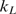 and 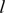 is advantageous. However, if the internal evolutionary dynamics are slow as is the case in Fig. 6 (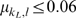), the vesicle-level selection can keep the evolving parasites at lower severity. This enables the internal replicator system to remain in the state in which the coexistence is deterministically stable (i.e. 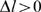); therefore, increasing 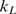 and 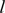 is advantageous.

Interestingly, Fig. 7 also reveals that the strength of the global flow correlates very well with the strength of the local flow around the unstable equilibrium for which only the replicase exists (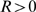 and 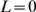). The latter is determined by the the speed at which the parasite invades an established population of replicases. Although unexpected, this correlation is not unreasonable: One the one hand, the extinction of a replicator system—the final outcome of the global flow—can be seen as the process in which the parasite replaces the replicase in the share of the total population which is shrinking to zero. On the other hand, the invasion of parasites into an “established” population of replicases—the local flow around the aforementioned unstable equilibrium—is also the process of population replacement. Therefore, there is a connection between the two processes. In contrast to this connection, the relationship between the strength of the local flow and the values of 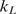 and 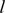 can be explained more concretely. For simplicity, let 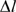 be some constant negative value for different values of 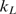 (the conclusion will be the same if we consider the values of 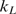 and 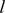 along the evolutionary trajectory). Let us consider Reaction 1(a). Since the population of the replicase has been established (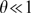), the limiting step for the parasite's invasion must be the reaction 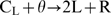. Thus, the speed of the invasion strongly depends on the equilibrium constant of the reaction 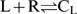 [i.e. 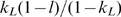]. Then, biasing the equilibrium towards complex dissociation (decreasing 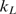) should slow down the invasion even though the effective association rate 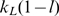 increases by the decrease of 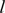 (since 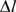 is constant), and vice versa.

### Analysis of the Surface Model

In this section, we show that the population dynamics of traveling waves exhibit the property of multiplication, variation and inheritance, and therby it ensures the macroscopic stability of the replicator system. Moreover, we analyze what kind of selection pressure exists among waves, which turns out to be qualitatively different from the vesicle-level selection that arises by default.

#### Short-term evolution

**Figure 8:**
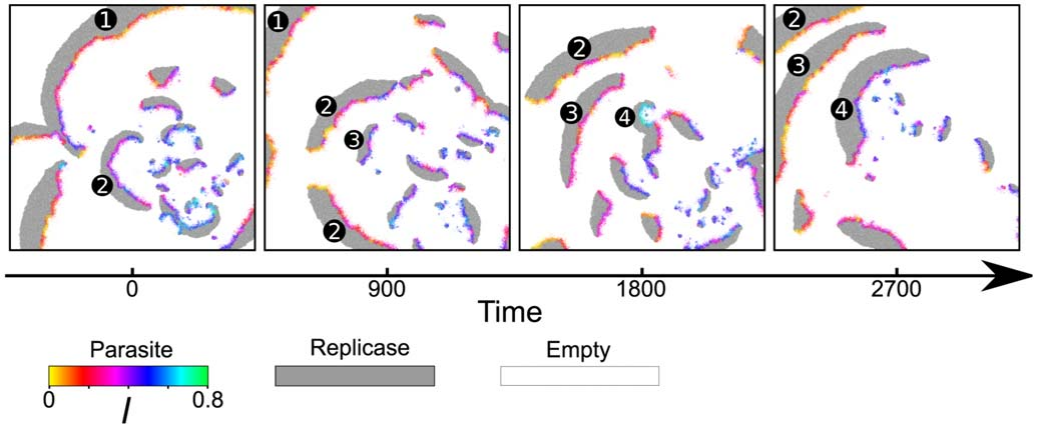
Life history of traveling waves. The figure shows consecutive snapshots of a simulation with fixed 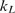 (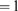). It depicts the aging and reproduction of traveling waves. The numbers depicted in the picture identify individual waves. The time was reset to zero when the first snapshot was taken. The size of the CA was 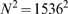 squares. The other parameters were the same as in Fig. 2 except 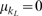 and 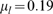 (initially 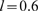).

As we did in the compartment model, we followed the dynamics of individual traveling waves to uncover the “life history” of waves. For simplicity, we took a case in which 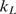 was fixed and set the parameters so as to put the system in a limiting situation wherein the long-term observation of individual waves was easier (see Fig. 8 caption). Fig. 8 shows consecutive snapshots of the simulation, and Video S3 depicts the same simulation starting at the first slide of Fig. 8. The results show the following.

Firstly, an individual wave changes its characteristics, as it travels, such that the parasite in the wave back evolves to decrease 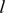 (the colors change from cyan to yellow). In other words, traveling waves experience a kind of “aging” in the form of the evolution of aggravated parasitism in a local replicator system. Comparable observation was made for vesicles too; however, the difference is also worth noting: Limited lifetime of a single wave is mainly due to the collision with other waves or boundaries, and therefore an isolated wave can, despite the aging, persist a far longer period of time than a single vesicle can (we will come back to this in the next section).

Secondly, new waves are generated mostly from the waves that consist of weaker parasites, i.e., those with greater values of 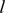 (blue or cyan). To understand this, we must first understand how new waves are generated. The generation of new waves typically begins with the isolation (or “escape”) of the replicase molecules which are originally at the front of an existing wave from the parasites which are at the back of the wave [28]. Two or more escaped replicase molecules can establish a new population, which is then “infected” by nearby parasites, resulting in a new traveling wave. Now, let us consider the effect of parasites' being weaker. Firstly, it makes the escape of replicase easier. Moreover, a newly generated wave is typically small and thus vulnerable to annihilation through the wave back “catching up” the wave front (i.e. the extinction of a local replicator system due to the over-exploitation by the parasites). This vulnerability is also circumvented if parasites are weaker. Therefore, waves with weaker parasites—more precisely, wave parts that have weaker parasites—can generate more new waves.

Thirdly, the parasites of a new wave descend from a small sub-population of the parasites of the wave that has generated the new wave. This is simply because of diffusion being finite and parasites replicating locally.

Finally, there is diversity in the population of parasites within a single wave (notice the color variation within each wave). This can be explained by finite diffusivity and stochasticity, which reduce the effect of local competition among parasites (cf. [37],[38]).

The last three points respectively allow selection, inheritance and variation in traveling waves. The resulting evolutionary dynamics of traveling waves counteract the evolution of too strong parasitism, and thereby, allow the macroscopic stability of the replicator system. Needless to say, the parallelism with the compartment model is striking.

Additionally, we note that the wave-level selection does not favor an unlimited weakening of parasites. This is because traveling waves generated by weaker parasites have smaller empty space in between, so that stronger parasites can “permeate” through the regions inhabited by too weak parasites and out-compete them (Video S4). Hence, the selection is stabilizing.

#### Long-term evolution

We have seen above that the surface model differs from the compartment model in an important aspect. Namely, a mesoscopic entity in the surface model (i.e. a traveling wave) can persist for a far longer time than that of the compartment model (i.e. a vesicle). This difference implies a shift in the focus of the selection, in that the wave-level selection tends to increase the fecundity of the wave rather than its longevity in contrast to the default vesicle-level selection (cf. [4],[39],[40],[41]). In this section, we first confirm this point and then investigate why the surface model and the modified compartment model, which both assume no functionality in the folded state of the parasite, results in the opposite evolutionary direction (Fig. 3).

**Figure 9:**
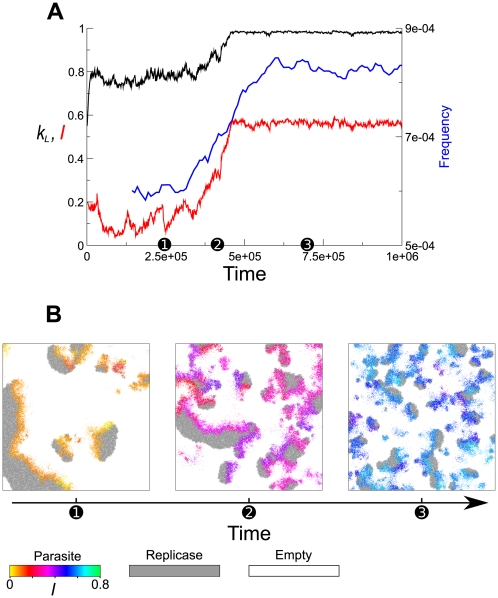
Evolutionary dynamics of traveling waves. **A:** The left y-axis is 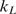 and 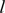 averaged over an entire population. The simulation was identical to that shown in Fig. 2 (surface model). The right y-axis is the frequency at which waves traverse the center of the grid (1/Time). Note that the width of time windows for the measurement of the frequency is quite large (ca. 28600), so that the curve is leveled off compared to that of 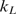 and 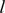. The details of the measurement are as follows. The number of replicators was measured in the region of 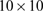 squares located at the center of the grid. At a certain moment, if the number of replicase molecules is more than zero, the moment was designated as on-state; otherwise, off-state. An off-on-off transition of the state was considered one event of wave traversing if the number of replicase molecules exceeds the background average during on-state. **B:** Snapshots of the simulation taken at the three time points designated on the abscissa of A. The size of traveling waves becomes smaller in concurrence of the increase of the frequency of wave traversing shown in A.

The evolutionary dynamics of the surface model are depicted from various aspects in Fig. 9. From the time plot of 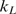 and 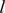, three time points were chosen that represent the initial, intermediate and final phase of the evolutionary dynamics (Fig. 9A, abscissa). For each of these time points a snapshot of the simulation is shown in Fig. 9B. The snapshots show that the size of waves became smaller, whereas the number of waves became greater. This observation was made more quantitative by measuring the frequency of waves traversing the center of the grid. As shown in Fig. 9A (blue line), this frequency increased in concurrence of the increase of 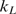 and 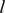. In addition to these results, we can deduce that the decrease of the wave size is a byproduct of the increase of the wave number from the fact that (1) the annihilation of waves is largely due to the collision with other waves, which becomes more frequent with the increase of the wave population, and that (2) waves must persist long enough to become large. In conclusion, the above analysis strongly indicates that the wave-level selection indeed operates on the generation of waves, i.e. the fecundity of waves.

To understand the factors influencing the fecundity of waves, we again analyzed the qualitative behavior of Eqn. 2. From Fig. 7 (right column) we can already glimpse such factors. For 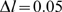, a population initialized with a small number of replicase and parasite molecules can grow and establish itself if the initial number of parasites is not too great; othwerwise, the population goes to extinct. Moreover, the phase area in which the trajectories lead to extinction (gray area) shrinks with the increase of 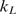 for 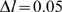. These results imply that increasing 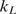 and 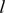 reduces the events of population establishment failure, which is likely to facilitate the establishment of new waves. However, there is a problem in this argument, in that if we consider the parameters relevant to the evolution in the surface model (e.g. Fig. 7 left column), all trajectory leads to extinction, so that there is little explanatory power in the qualitative phase portrait for such a parameter.

**Figure 10:**
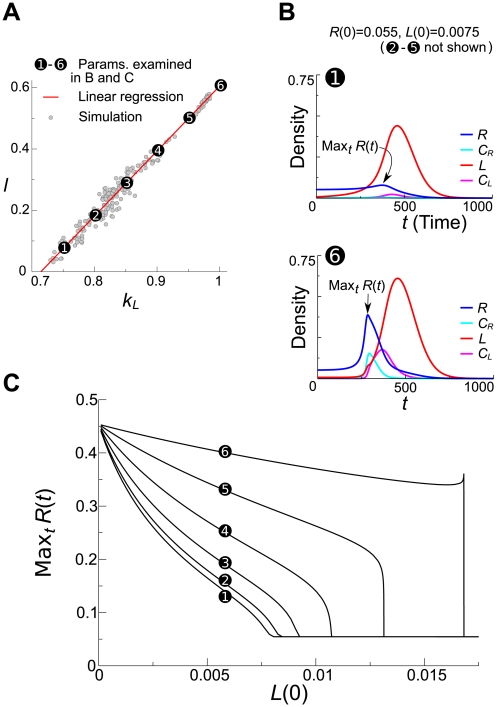
Analysis of transient dynamics of the ODE model (Eqn. 2) related to the establishment of new waves. **A:** The evolutionary trajectory of 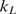 and 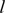 in the surface model. From this trajectory, we obtained the parameter sets used in the analysis of the ODE model shown in B and C (the numbers depicted in A, B and C correspond to each other). For this sake, the initial condition was changed (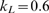 and 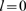; otherwise, the parameter setting was identical to that in Fig. 2. Note that linear regression underestimates the slope due to the error in abscissa, which results in the stronger underestimation of 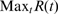 shown in B and C for greater 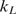 and 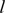. **B:** Numerical solutions of Eqn. 2. The values of 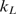 and 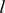 were as indicated in A. The other parameters were the same as in Fig. 1. The initial condition was as follows: 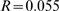; 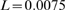; 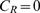; 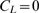. **C:** The maximum value of 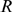 from transient dynamics of Eqn. 2 as depicted in B is plotted as a function of the initial value of 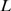 (otherwise, the initial condition was the same as in B).

Therefore we next analyzed the transient behavior of Eqn. 2 for the parameters chosen from the evolutionary trajectory (Fig. 10A). The vital point of this analysis was that the initial condition was set so as to mimic the situation in which a new wave is generated; i.e., 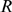 was set slightly above the minimum value required to surmount the Allee effect, and 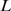 was set to very small values, and 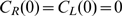. Fig. 10B shows numerical solutions of Eqn. 2 with different parameter sets for an identical initial condition. The time plots show that if 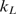 and 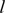 are smaller (i.e. earlier in the evolution), the growth of 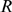 is quickly obliterated by the outbreak of 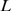, whereas if 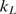 and 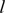 are greater (i.e. later in the evolution), 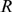 can grow up to quite a high value before extinction. We then calculated the maximum value of 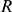 as a function of 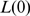 shown in Fig. 10C. The results show that the maximum values of 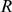 is greater when 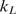 and 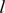 are greater (i.e. later in the evolution), and the difference expands as 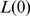 increases. These results strongly indicate that increasing 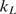 and 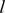 enhances the transient growth of a replicase population in the presence of small number of parasite molecules and, thereby, should enhance the establishment of new waves in the surface model.

Having identified the link between the wave generation in the surface model and the transient behavior of the well-mixed replicator system, we can now intuitively understand the relationship between the wave generation and the parameters 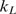 and 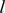. For simplicity, let 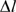 be some constant negative value for different values of 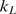 as we did before in the compartment model. The focal process is the competition between the transient growth of 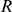 and the growth of 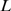. Since 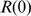 and 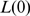 are small (i.e. 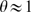), the limiting process for the growth of 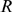 and that of 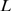 must be the complex association, i.e., 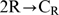 and 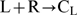, respectively. Then, decreasing the effective rate of the latter reaction, 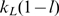, should ease the growth of 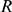 by releasing the competition even though the equilibrium of the association-dissociation reaction between parasites and replicases is biased more to the association (since 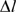 is constant). This argument is supported by the time plot shown in Fig. 10B, in that the increase of 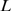 is slower for the greater value of 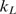 and 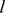.

### Summary: Comparison between the Explicit and Implicit Multilevel Selection Models

• The two multilevel selection models are quite similar in how they achieve the macroscopic stability of the replicator system: the evolutionary dynamics on the microscopic entities (i.e. replicators) are counteracted by the evolutionary dynamics on the mesoscopic entities (i.e. vesicles or traveling waves).

 - In the compartment models, the vesicle-level selection operates on the variability in internal replicator systems generated by the stochastic evolutionary dynamics of replicators.

 - In the surface model, selection operates on the level of traveling waves, which have the feature of multiplication, variation and inheritance.

• However, the two types of mesoscopic entities differ in their stability in isolation.

 - In the compartment model, a vesicle is an externally imposed mesoscopic entity (explicit multilevel selection), and it is less persistent.

 - In the surface model, a traveling wave pattern is a self-organized mesoscopic entity (implicit multilevel selection), and it is thus more persistent than a vesicle (if it is too unstable, it would not self-organize).

• The difference in the stability of mesoscopic entities results in the difference in the focus of mesoscopic selection.

 - The vesicle-level selection, by default, operates for the longevity of vesicles due to its greater instability.

 - The wave-level selection operates for the fecundity of waves (i.e. the generation of new traveling waves).

• Because multilevel selection keeps the evolution of too severe parasitism at bay, parasites have a trade-off situation between 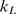 (i.e. affinity to replicase) and 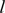 (i.e. template availability). Under this trade-off, parasites can adopt two kinds of strategies in the association-dissociation reaction with replicase: (A) increasing the effective rate of association—smaller 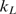 and 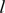
—and (B) biasing the equilibrium towards association—greater 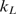 and 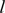. Strategy A weakens the deterministic flow of the replicator dynamics while prohibiting the transient growth of a population consisting of a small number of replicases and parasites. Strategy B strengthens the deterministic flow while enhancing such transient growth.

These strategies gain selective differences through the interactions between the dynamics of microscopic entities and those of mesoscopic entities. This produces a novel trend in the long-term evolution of the replicator system, which can differ between the two multilevel selection models.

 - In the compartment models, the death rate of vesicles depends on the stability of the coexistence between the replicase and parasite in the internal replicator system. If the coexistence is deterministically stable, strengthening the deterministic flow of the internal replicator dynamics is favored. If the coexistence is deterministically unstable, weakening the deterministic flow is favored.

The evolutionary dynamics of internal replicator systems are fast when the mutation rate of replicators is high and the population size of internal replicator systems is large. In this case, the coexistence is likely to be deterministically unstable; therefore, weakening the deterministic flow of the replicator dynamics (i.e. Strategy A) is favored. (Similarly, if the internal replicator evolutionary dynamics are slow, Strategy B is favored.)

However, this default direction of the vesicle-level selection can be overruled by an additional selection pressure arising from the (predefined) functionality of the folded state to facilitate the vesicle growth.

 - In the surface model, the establishment of new waves depends on the (transient) growth of a population consisting of a small number of replicases and parasites. Therefore, Strategy B is favored.

### Diffusion Rate and Vesicle Volume: Important Parameters of Multilevel Selection

In this section, we compare the surface model and the compartment model with respect to how the macroscopic stability responds to the change of either the diffusion rate (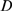) in the surface model or the threshold volume for division (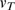) in the compartment model, whereby we illustrate an interesting difference between the two models. Our choice to focus on 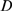 and 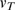 is based on two reasons. Firstly, previous studies have suggested that these parameters significantly affect the macroscopic stability of the replicator system [11], [28]–[30],[33],[42],[43]. Secondly, 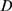 and 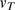 play a similar role in how they limit the macroscopic stability; i.e., increasing 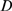 and increasing 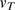 increase the number of replicators involved in local replicator systems that can be considered independent of each other due either to vesicle boundaries or to spatial distance (one traveling wave can be considered to consist of multiple such local replicator systems as seen from the heterogeneity within a wave shown in Fig. 8).

**Figure 11:**
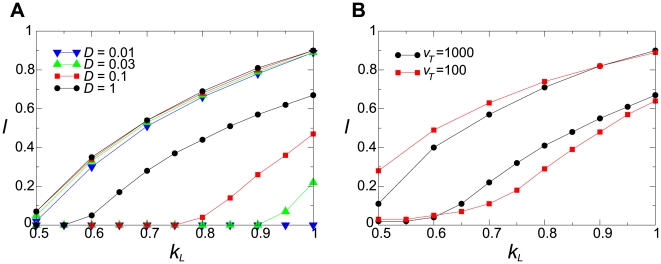
Survival region in the parameter surface of 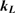 and 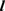. **A:** The surface model. The upper four lines, which are largely overlapping, designate the upper boundaries of the survival regions. The lower four lines designate the lower boundaries. The mutation was disabled (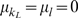). 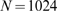 (CA size). The other parameters not depicted in the graph are the same as in Fig. 2. In addition, we note that the root mean square displacement of a molecule in its life time was about 10 squares for 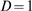. **B:** The vesicle model. The upper two lines designate the upper boundaries of the survival regions; the lower two lines designate the lower boundaries. Note that lower boundaries are influenced by the fact that the growth of target volume depends on 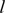 (i.e., 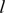 is bounded below because 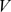 depends on 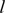 and 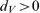), which is not the case in the surface model. To reduce this influence, the decay rate of target volume is reduced to 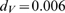 (using the modified compartment model avoids the problem better; however, it did not matter for our conclusion as shown in Fig. S1). 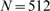 (note that 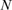 can be smaller in the compartment model than in the surface model in order to remove the influence of the smallness of the system because vesicles are smaller than waves). The other parameters not depicted in the graph are the same as in Fig. 2.

Firstly, we investigated what might be called the “ecological” stability of the system, i.e. the range of 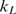 and 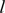 for which the replicator system exhibits the macroscopically stable coexistence of the replicase and parasite with no mutation (i.e. 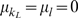). As seen from Fig. 11, while the survival regions from the two models are similar in topology, they significantly differ in its response to the change of 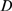 or 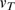. That is, the survival area in the surface model greatly varies as 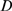 changes whereas that of the compartment model varies relatively little as 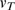 changes (the modified compartment model showed qualitatively the same result; see Fig. S1). Additionally, we mention that the compartmentalized system was not viable for 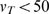, which was most likely because of too great assortment load [29],[42]. Also, the spatial pattern in the surface model appeared different for large diffusion rates as shown in Video S5 (see Text S1 for more on this point).

**Figure 12:**
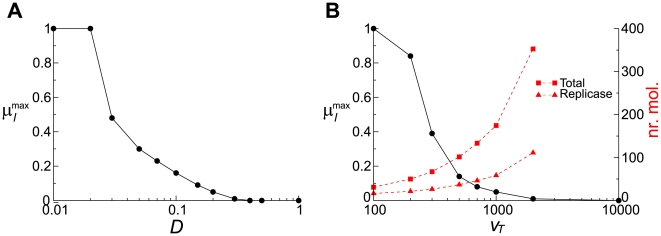
Maximum mutation rate (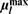) for which the system can survive. For simplicity, 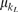 was set to 0. 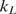 was set to 1. **A:** The surface model. The abscissa is diffusion rate. Note that a steep increase in 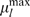 as 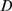 decreases from 0.03 to 0.01 is due to the fact that the system survives for (almost) any values of 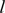 as seen from Fig. 11A. 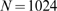 (CA size). The other parameters not depicted in the graph were the same as in Fig. 2. **B:** The compartment model. The abscissa is the threshold volume for division. The left y-axis is 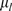; the right y-axis is the average number of molecules inside a vesicle: the squares denote the total number of molecules; the triangles denote the number of replicase molecules. A complex molecule is counted twice depending on the composition; e.g., 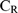 is counted as two molecules of 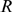. 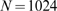. The other parameters not depicted in the graph were the same as in Fig. 2.

Secondly, we investigated the “evolutionary” stability of the system, i.e. the maximal tolerable value of 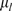 (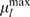) for which the system exhibits the macroscopically stable coexistence of the replicase and parasite (with 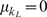 and 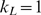) as shown in Fig. 12 (the modified compartment model showed qualitatively the same result; see Fig. S2). The result shows that 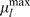 significantly changes as the parameter varies in both models, and the curves of 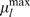 are very similar to each other. These results are in stark contrast to the response of the survival regions seen above.

These results indicate that there are two different aspects in the macroscopic stability of replicator systems. One is the range of rate constants for which a system displays the macroscopic stability—“ecological stability”. The other is the degree of perturbation to rate constants (i.e. mutation in the sense used here) for which a system displays the macroscopic stability—“evolutionary stability”. Interestingly, these two aspects do not necessarily correspond to one another. Consequently, the two models showing different degrees of ecological stability can show similar degrees of evolutionary stability.

Fig. 11 and 12 depict the general tendency that the macroscopic stability decreases as either 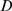 or 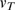 increases. This is explained by the fact that increasing the number of replicators involved in the local replicator systems reduces the stability of the whole system in two ways. Firstly, it decreases the stochasticity in the dynamics—both ecological and evolutionary—of the local replicator systems, which diminishes the relative impact of higher-level selection through reducing the variability among the local systems [9] (in the surface model, the local variability referred here also includes the heterogeneity within each wave). Secondly, it increases the frequency at which stronger parasites appear through mutation in a local replicator system per unit time [29],[44], which speeds up the evolutionary dynamics of local replicator systems (see also the caption to Fig. 6; note that this is irrelevant to the ecological stability by definition). It is worth mentioning that the expansion of the survival region by decreasing 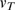 decreases as 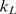 increases as shown in Fig. 11B and S1B (see the survival range of 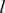 as a function of 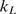). This is nicely explained by the first effect explained above and the fact that the deterministic flow of the replicator dynamics strengthens as 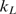 increases. Additionally, we note that the fraction of replicases within vesicles increases as 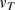 decreases. A similar observation has been made in other models [30],[45],[46] (esp. [45])

However, as we saw above, the survival area of the surface model is far more sensitive to 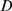 than that of the compartment model is to 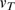 (Fig. 11). This can be explained by another effect of changing 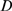; that is, decreasing 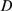 makes it more difficult for a parasite molecule to be in contact with replicase molecules than for a replicase molecule (see [43] for more quantitative investigation on this). This means that decreasing 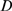 directly disadvantages the parasite. Therefore, the survival area substantially changes as a function of 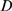. However, despite this additional effect, the dependency of 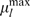 of the surface model is not necessarily more sensitive to 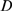 than that of the compartment model is to 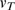 (Fig. 12). This is explained by the fact that the ecological stability is “offset” by the evolutionary dynamics; i.e., for greater mutation rates the replicator system tends to go to the lower boundary of the survival region through evolution (it is assumed that the ecologically stable region does not cover the entire span of the parameter range; see the case of 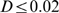 in Fig. 12A).

To summarize, the macroscopic stability of the interacting replicator system has two different aspects: ecological stability and evolutionary stability. In the surface model, decreasing 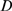 substantially enhances the ecological stability because decreasing 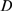 directly decreases the chance of parasites' being in contact with replicases. Contrastingly, in the compartment model, decreasing 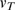 does not have such a direct effect; hence, its enhancement of the ecological stability is more moderate. However, this apparent advantage of the surface model with respect to the ecological stability is offset by the tendency of the evolutionary dynamics to bring the system to an edge of the ecologically stable parameter region, which indicates the greater importance of the evolutionary stability relative to the ecological stability. Consequently, the two models display a similar response in the evolutionary stability as a function of 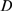 or 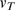.

## Discussion

The current study compared two multilevel selection models of replicator systems that had identical microscopic entities, but had qualitatively different mesoscopic entities. Despite the difference in the mesoscopic entities, we found that the two models were quite similar in how they achieved the macroscopic stability of the replicator system. Moreover, we also discovered an emergent trade-off situation in microscopic entities, which arose due to the multilevel selection (we note that a similar trade-off situation was previously discovered by van Ballegooijen and Boerlijst [41]). Interestingly, under this trade-off situation, microscopic entities displayed novel long-term evolutionary trends, which originated from the interactions between the dynamics of microscopic entities and mesoscopic entities. Furthermore, in contrast to the similarity mentioned above, the two models could sharply differ in the direction of this evolutionary trend, which was explained in terms of the difference in the stability between self-organized mesoscopic entities and externally imposed mesoscopic entities.

The surface model showed that the parasite, through long-term evolution, increased the time it spent in the state in which it could not function as template, despite the fact that no functionality was predefined for this state. Since the folding of an RNA molecule is likely to reduce the template activity, this result can be interpreted as the implication that in the diffusion-limited surface-bound system the parasite can evolve stable folding “for free”, i.e. without any specific functionality in the folding. The evolution of stable folding might be used as substrate for the further evolution of new functionality. Hence, the current study revealed a novel advantage of spatial self-organization for the evolution of complexity in RNA-like replicator systems.

In the compartment model, we found a simple relationship between the persistency (i.e. longevity) of a vesicle and the dynamical property of the replicator system inside the vesicle. That is, if the evolutionary dynamics of internal replicator systems are fast, the coexistence of the replicase and parasite in internal replicator systems is deterministically unstable; hence, weakening the deterministic flow of the internal replicator dynamics would increase the longevity of vesicles. Similarly, if the evolutionary dynamics of internal replicator systems are slow, the internal replicator coexistence is deterministically stable; hence, strengthening the deterministic flow of the internal replicator dynamics would increase the longevity of vesicles. This point seems to be generally relevant in compartmentalized interacting replicator systems (i.e. the systems where replicases catalyze the replication of templates).

The crucial difference between this study and our previous study [30] lies in the type of replicator systems considered: the current study considered a compartmentalized interacting replicator system, whereas the previous study considered a compartmentalized non-interacting replicator system (i.e. the systems where templates self-replicate). Since the evolutionary dynamics of the non-interacting replicator system is, in principle, stable under well-mixed conditions [13],[26],[27], the death of vesicles hardly happened unless externally introduced in the previous study. Therein we reported that it was essential to set the death rate of vesicles—which was one of the parameters—sufficiently high for the vesicle-level selection to be effective. In contrast, vesicle death in the current model was not only an internal—or spontaneous—process due to parasites (i.e. no need to externally introduce vesicle death), but also more frequent to those that contain more severe parasites, which reinforces the vesicle-level selection. Interestingly, therefore, parasites, which were the very reason the higher-level selection had to be considered, actually made the vesicle-level selection more effective.

We add that we deliberately avoided making a quantitative comparison between the models with respect to the area of the survival region in the parameter space and the maximum tolerable mutation rate for the following reasons. Firstly, the models have qualitatively different kinds of selection pressure because of the functionality of the folded state of parasites. Secondly, the result of a quantitative comparison depends on the parameters (e.g. one model can have a greater or smaller value of 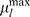 depending on the value of 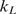). Thirdly, the models do not have a completely identical set of parameters (most prominently, it is unclear how to scale 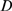 and 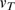). Fourthly, there are two different kinds of population size, i.e. that of microscopic entities and that of mesoscopic entities, which can change through evolution (e.g. Fig. 9). These points make the definition of fairness in quantitative comparison impracticable. Therefore, we concentrated on the qualitative comparison.

We should also mention an important simplification made in the current models; i.e., mutations of replicators were restricted to the perturbation of the two parameters of parasites. Other types of mutation processes can have significant impacts on the eco-evolutionary dynamics of replicator systems. The diversity in the replicase population [30] and/or deleterious mutations [28] can disadvantage parasites by effectively “diluting” the replicase population. Moreover, the explicit consideration of genotype-phenotype-interaction mapping allows a positive feedback in the evolution of these three levels, which stabilizes the whole system [35]. Hence, subjecting a greater degree of freedom to evolution seems to have positive effects on the stability of the replicator systems. In the current study, however, we restricted these processes for a clear-cut elucidation of the effects of different multilevel selection mechanisms.

Finally, let us comment on an interesting difference between the modern cell and the protocell conceived in this study (i.e. the vesicle containing replicators). The difference lies in the concept of genotype, which, we commonly assume in evolutionary biology, is a static state of an individual. Such an assumption can be justified for a modern cell because of the small rate of somatic mutation relative to the lifetime of the cell. However, it is clearly invalid for the protocell in the current study because the internal replicator system—of which population composition can be considered as the “genome” of the protocell—greatly changes its state over time comparable to the lifetime of a vesicle (Fig. 4). Stated differently, one cannot separate from each other the timescale of the eco-evolutionary dynamics of the microscopic entities and the population dynamics of the mesoscopic entities. It would be an interesting future project to investigate how these two timescales can be split apart through evolution (see also []).

There are ongoing efforts to synthesize chemical systems that can undergo self-sustained Darwinian evolution in the laboratory. In particular, Szostak *et al.* have been making steady progress towards the laboratory synthesis of model protocells[31], [32], [49]–[53]. In these studies, the diameter of vesicles ranged from 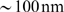 to 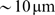. Assuming that the vesicle dynamics can keep pace with the replicator dynamics, the number of replicators inside the vesicle should be about 100 to counter the evolutionary instability of the kind of replicator systems investigated in this study (Fig. 12B). Thus, the concentration of polynucleotide inside the vesicle should be 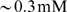 for 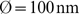 and 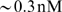 for 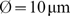, where it is assumed that vesicles are spherical and unilamellar (for multilamellar vesicles, the greater concentration would be allowed). Next, there are experimental techniques to amplify polynucleotide in diffusion limited media, the so-called “molecular colony” or “polony” technology [54],[55]; and its possible use for chemical Darwinian systems has been suggested [56]. Under the condition in which this technique is normally practiced, it can be calculated that within a molecular colony a volume of 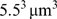 contains about 4000 polynucleotide molecules of about 100 bases and that it takes about 10 sec [which we assume is a generation time (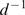) of replicators] for diffusion to displace a molecule of a similar length by 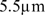 (see also Text S1 note 6). The corresponding number of molecules is about 10 for 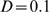 (100 for 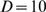) in the current surface model. Thus, to obtain a similar situation under the molecular colony technology, either the concentration within a colony must be reduced to 0.1 nM or the diffusion constant must be reduced to 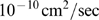 (but see Text S1 note 7). Unfortunately, these figures are not applicable to the best currently available RNA-directed RNA polymerase ribozyme (a candidate of the replicators investigated here), because it is at best three order of magnitude slower polymerase than the protein polymerases used for PCR [57]. Moreover, mineral surfaces have been suggested to have various chemical advantages for the origin of life (e.g. [49],[58],[59],[60]). Besides chemical aspects, it appears to us that mineral surfaces also have an advantage in the current context by confining the replicator in two-dimensional space. However, to our knowledge, experimental data necessary for the type of calculation made above seem to be not yet available. Finally, Koonin and Martin recently discussed a system compartmentalized by inorganic boundaries which are static relative to the internal replicator dynamics [61] (it is reported that related experimental work is in progress [62]). The current model can easily be extended to simulate a simplified version of such a system by disabling the growth and division of vesicles and by allowing the small diffusion of replicators across compartment boundaries. Our preliminary investigation showed that a model with static compartments displayed the formation of the large traveling waves typically spanning more than 10 compartments (where 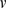 was fixed either to 100 or to 1000, and 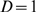 inside compartments, and 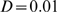 across compartment boundaries), which gives rise to the evolutionary dynamics on the level of waves. Thus, interestingly, it is spatial self-organization that plays an important role for the macroscopic stability of the static compartment model, despite the fact that the system is explicitly compartmentalized.

Traditionally, multilevel selection has been investigated in the context of altruism-egoism dichotomy. In this context, models are constructed by defining the traits (or strategies) of individuals directly with respect to its fitness contributions at different levels of biological organizations either through a priori conception or through inference from observation as such (e.g. [63], for review; see also Text S1 note 8). By using these models, the classical theory asserts that a trait that disadvantages an individual can still evolve if it advantages certain higher-level biological organizations. By contrast, the models in the current study were constructed without preconceiving what is costly or beneficial on what level of organization (see also Text S1 note 9). Therewith, this study—while concordant with the classical theory—gives two novel implications. Firstly, interactions between the dynamics of microscopic and mesoscopic entities can generate novel evolutionary directions (or strategies) not conceived in the altruism-egoism dichotomy. Secondly, difference in mesoscopic entities can lead to difference in the long-term evolutionary trend of otherwise identical microscopic entities. Hence, we suggest that it is necessary to go beyond the classical modeling framework in order to explore a possible plethora of novel evolutionary directions—beyond that found here and in [41],[43]—that can be generated through multilevel evolution.

## Supporting Information

Text S1 This file consists of the following sections: (1) additional notes to the main text; (2) the extension of Equation 2 with two parasite species; (3) the details of the replicator CA model; (4) the details of Cellular Potts Model (CPM) and coupling between CPM and the replicator CA model; (5) Behavior of the surface model for greater diffusion rates (Figures S3 and S4).
(0.53 MB PDF)

Figure S1 Survival region in the parameter surface of *k_L_* and *l* in other settings than in Figure 11. A: The survival region of the compartment model with a greater decay rate of target volume. *d_V_* was set to 0.06 (the same value as used in the evolution simulation). B: The survival region of the modified compartment model, where the target volume *V* was fixed. For *v_T_* = 100, *V* = 140; for *v_T_* = 1000, *V* = 1400. In A and B, the parameters not shown in the figure were the the same as in Figure 11B. Figure S1 shows that the survival regions are similar between the compartment model with *d_V_* = 0.006 (Figure 11B) and the modified compartment model (Figure S1B). Moreover, the survival region of the compartment model with *d_V_* = 0.06 (Figure S1A) is smaller than that of the modified compartment model (Figure S1B). This is because the faster decay of the target volume (greater *d_V_*) has to be compensated by the greater growth of it (greater *l* and/or greater *L*). As a corollary, one can also understand the result that, for greater values of *k_L_*, the two compartment models display a similar lower boundary of *l*. The above results indicate that if *d_V_* is sufficiently small or *k_L_* (*l*) is sufficiently great, the survival region of the original compartment model is mostly determined by the stability of internal replicator systems rather than by the growth of the target volume.
(0.21 MB EPS)

Figure S2 Maximum mutation rate (*μ_l_* ^max^) for the modified compartment model. *μ_l_* ^max^ is plotted as a function of the threshold volume for division (*v_T_*). In the modified compartment mode, the target volume *V* was fixed (*V* = 1.4*v_T_*). The parameters not shown in the graph were the same as in Figure 12. Figure S2 shows that the values of *μ_l_* ^max^ are similar to those from the original compartment model with *d_V_* = 0.06 (Figure 12).
(0.11 MB EPS)

Video S1 Compartment model dynamics. A movie of an early part of an evolution simulation with the compartment model. The parameters were the same as in Figure 2 except that the size of the CA was reduced to *N* = 256 for visibility. The upper left panel depicts the CA by the value of *l*. The color coding is the same in Figure 2. The upper right panel depicts the CA by the value of *k_L_*. The color coding is the same as in the histograms, which are explained below. The lower panels are as follows: the first panel from the left depicts the frequency distribution of the average value of *l* of vesicles; the second panel from the left depicts the frequency distribution of the average value of *k_L_* of vesicles; the third panel from the left depicts the frequency distribution of the value of *l* of parasites; the forth panel from the left depicts the frequency distribution of the value of *k_L_* of parasites. In every histogram, the abscissa ranges from 0 to 1 with 100 bins, and the ordinate ranges from 0 to 0.3. The time is from 0 to 19826 (see the legend of Figure 4 for the timescale).
(10.00 MB MPG)

Video S2 Surface model dynamics. A movie of an early part of the evolution simulation with the surface model depicted in Figure 2. The color coding is the same as in Figure 2. The time is from 0 to 62261.
(9.18 MB MPG)

Video S3 Life history of traveling waves. A movie of the simulation depicted in Figure 8. The color coding is the same as in Figure 8. The histogram appearing in the upper left corner of the movie depicts the frequency distribution of the value of *l* of parasites, where the abscissa ranges from 0 to 1 with 100 bins, and the ordinate ranges from 0 to 0.1. The color coding of the histogram corresponds the color of parasites. The time is from 0 (the first snapshot of Figure 8) to 339722.
(7.98 MB MPG)

Video S4 Competition between parasites. A movie of a competition experiment with the surface model. The movie shows that there is stabilizing selection on the strength of parasitism (the value of *l* in this case). The movie depicts that weaker parasites (*l* = 0.7 and *k_L_* = 1; initially placed in the right half of the simulation field; the color is cyan) were out-competed by stronger parasites (*l* = 0.55 and *k_L_* = 1; initially placed in the left half of the field; the color is blue) through the “permeation” of the stronger parasite into the region occupied by the weaker parasite, which happens because of too small empty space between the traveling waves made of the weaker parasite. The time is from 0 to 17751.
(2.61 MB MPG)

Video S5High diffusion case in the surface model. A movie of a simulation with the surface model for a greater diffusion rate (*D* = 1). The color coding is the same as the other movies and snapshots of the surface model. The mutation was disabled 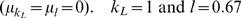 (the value of *l* was chosen to be just above the lower survival boundary shown in Figure 11A). *N* = 1024 (CA size). The other parameters were the same as in Figure 11A. See also an explanation in Text S1. The time is from 0 to 34650.(3.66 MB MPG)
