# Supplementary material for: Multilevel Selection in Models of Prebiotic Evolution II: A Direct Comparison of Compartmentalization and Spatial Self-Organization
Source: PLoS Comput Biol. 2009 Oct 16;5(10):e1000542. doi: 10.1371/journal.pcbi.1000542 (PMC2757730; doi:10.1371/journal.pcbi.1000542)
Supplement: Text S1 — This file consists of the following sections: (1) additional notes to the main text; (2) the extension of Equation 2 with two parasite species; (3) the details of the replicator CA model; (4) the details of Cellular Potts Model (CPM) and coupling between CPM and the replicator CA model; (5) Behavior of the surface model for greater diffusion rates (Figures S3 and S4). (0.53 MB PDF) [file pcbi.1000542.s001.pdf]

Text S1—Supporting Information for "Multilevel Selection in Models of Prebiotic Evolution II: a Direct Comparison of Compartmentalization and Spatial Self-Organization"

Nobuto Takeuchi[1*], Paulien Hogeweg[1],
1 Theoretical Biology/Bioinformatics Group, Utrecht University, Utrecht, the Netherlands
∗ E-mail: Corresponding takeuchi.nobuto@gmail.com

Notes

The main text refers to the following additional notes.

1. This is often called information-threshold or error-threshold problem; however, the problem itself is actually independent of the existence of threshold-like behavior (i.e. abrupt transition) in the replicator dynamics [1].

2. We also studied a case without this assumption, where the sum of the folding rate and unfolding rate equaled to κ. The results were essentially the same as explained in this paper.

3. k_L and l are bounded in [0,1], where we used reflecting boundary so that, without selection, mutations generate uniform distribution. To be more precise, let k_L be originally x, and y be drawn from $[-\delta/2, \delta/2]$. If $x + y < 0$, k_L becomes $-x - y$; if $x + y > 1$, k_L becomes $2 - x - y$.

4. It is possible to set the size of vesicles and diffusion inside vesicles so as to allow the formation of traveling waves within vesicles. However, such a system is better considered a hybrid of the models investigated in this study. Because the purpose of the current study is to compare two different mechanisms of multilevel selection, we do not investigate such a hybrid model.

5. We also considered the stability of the the equilibrium with extinction (i.e. $R = L = C_R = C_L = 0$, which is always locally stable due to the Allee effect [2]), since if this equilibrium were less stable, the replicator coexistence might be relatively more stable. However, this line of analysis was also unsuccessful for the same reason as before.

6. The diameter of a colony (sphere) is about 200 μm for a template of 120 bases in 15% acrylamide gel (5% bis) [3]. We assumed that the amplification factor of PCR is 10^8 [4]. Assuming uniformity within a colony, the concentration of polynucleotide is about 40 nM. We assumed that the diffusion constant of ssDNA of 120 bases is 5×10^{-9} cm^2/sec in the same gel at 55°C [5]; hence, the root mean square displacement ($\sqrt{6Dt}$) is about 5.5 μm in 10 sec. Moreover, it is assumed that the generation time is 10 sec, given the speed of polymerization ranging from 30 nt/sec (Qβ replicase [6]) to 110-300 nt/sec (*Taq pol*/KOD polymerase [7]).

7. There are two aspects in the influence of diffusion on the replicator dynamics: the number of distinct replicators one replicator can potentially interact with; the frequency at which a replicator interacts with any replicators. The former is related to the spatial correlation between replicators and has been investigated in this study, whereas the latter is related to the Allee effect [2], and the current models have implicitly assumed that it causes not much problems by assuming a high density of replicators within immediate interaction distance (cf. [8]). In the main text, the reduction of the concentration or the diffusion constant of templates was suggested by considering the former aspect; however, there is thus a caveat in that suggestion because the latter aspect can make a system inviable in reality.

8. For example, in the classical model of group selection, trait X is defined by its fitness effect a_X to its carrier and by its fitness effect b_X to the other individuals [9]. X is considered either altruistic or egoistic depending on the value of a_X and b_X.

9. In the current model, we could consider, e.g., having a great value of k_L and l as a "trait". However, not being explicitly defined by the model, what selective (dis)advantage such a trait brings to what entity had to be discovered by analyzing the replicator dynamics at multiple levels.

Extension of Eqn. 2 to a System with Two Parasite Species

$$
\begin{aligned}
\dot{R} &= -2k_R R^2 + [2(1-k_R) + 3\kappa\theta + 2d]C_R + \sum_{i=1}^{2}\{-k_{L_i}RL + [(1-k_{L_i}) + \kappa\theta + d]C_{L_i}\} - dR,\\
\dot{C}_R &= k_R R^2 - [(1-k_R) + \kappa\theta]C_R - 2dC_R,\\
\dot{L}_i &= -k_{L_i}(1-l_i)RL_i + [(1-k_{L_i}) + 2\kappa\theta + d]C_{L_i} - dL_i,\\
\dot{C}_{L_i} &= k_{L_i}(1-l_i)RL_i - [(1-k_{L_i}) + \kappa\theta]C_{L_i} - 2dC_{L_i},
\end{aligned}
$$

where i denotes different species of parasites; and $\theta \equiv 1 - R - 2C_R - \sum_i [L_i + 2C_{L_i}]$. All numerical calculations in this study were done by using either GRIND [10] or CONTENT [11].

Details of the Replicator CA Model

The dynamics of Reaction 1 was modeled in stochastic cellular automaton (CA) framework. The model is a spatially extended, individual-based, Monte Carlo simulation model. It consists of a two-dimensional square grid and molecules located on the grid. One square in the grid can contain at most one molecule, which is either R or L; empty square is considered as the generalized resource for replication (θ in Reaction 1). A complex molecule is represented by two molecules that have an appropriate "flag" and are located in contiguous squares, where contiguity is defined as the eight nearest squares (Moore neighbors); hence, C_R consists of two R molecules, and C_L consists of one R and one L molecule. The state of the CA is fully specified by the spatial distribution of molecules with flag information. The temporal dynamics is run by consecutively applying the stochastic algorithm that simulates Reaction 1 and diffusion.

The reaction-diffusion algorithm effectively runs as follows:

1. Randomly choose one square from the plane (every square with an equal probability).

2. Randomly choose one of the eight nearest squares of the square chosen first. Depending on the contents of the two, the reactions that can happen, the possible reactions, are determined (including teh first-order reactions such as decay). For example, if the two molecules are parasites (L), the complex formation reaction is not included in the possible reactions.

3. Execute one of the possible reactions with a probability calculated as the reaction rate constant multiplied by a common constant α. The value of α is chosen such that the maximum possible value of the sum of the probabilities of the possible reactions is less than 1.

Diffusion is considered as a second-order reaction with rate constant D (which is proportional to the macroscopic diffusion constant), and it is implemented by swapping the content of two squares. If complex molecules diffuse, swapping is done so as to maintain the association of molecules (see below). Diffusion and reaction across the grid boundaries are prohibited (no-flux boundary condition). The application of the above algorithm for N^2 times, where N^2 is the number of squares in the grid, is arbitrary defined as one time-step of replicator dynamics.

The algorithm described above was obtained by slightly modifying one described in [12], wherein it was shown that the dynamics under that algorithm approached that of the Gillespie algorithm when $D \to \infty$ with N being constant. The current modification (see below) preserves this property and enhances the applicability of the algorithm to a situation wherein the number of possible higher-order reactions is huge

because of great diversity in the kinds of molecules. The C++ program that implements the current model is available from the authors upon request.

The details of diffusion algorithm involving complex molecules are as follows. There are three possible cases in which swapping involves a complex molecule: (1) one molecule is forming a complex with some molecule, and the other is not; (2) the two molecules are forming a complex with each other; and (3) each molecule is forming a complex with other molecules. In case (1), let x and y be the squares chosen for diffusion (it does not matter which is chosen first), and let us suppose that x contains a non-complex molecule or is empty, and y contains a complex molecule. Let y' be the square containing the molecule with which the molecule in y is forming a complex. Then, swapping is done as follows: the molecule in x is moved to y'; the molecule in y is moved to x; the molecule originally in y' is moved to y. In case (2), the two molecules swap their position (i.e. the rotation of the complex molecule). In case (3), let us suppose that x also contains a complex, and let x' be the square containing the molecule which which the molecule in x is forming a complex. Then, swapping is done as follows: $x \to y'$; $x' \to y$; $y \to x'$; $y' \to x$, where arrows mean that the molecule originally in the left square will be moved to the right square.

The details of the aforementioned modification are as follows. In the previous algorithm, the reaction that happened to the molecule in the first chosen square was determined before choosing the second square. Firstly, all possible second-order reactions the molecule in the first square could experience were included in the possible reactions. Secondly, the probability of each possible reaction was calculated by multiplying α to its rate constant. Thirdly, according to these probabilities, one reaction was chosen. Fourthly, if the chosen reaction was a second-order reaction, a neighboring square was chosen. Finally, if the neighboring square contained the right type of molecules for the reaction to happen, the reaction actually happened; otherwise, nothing happened. In the current algorithm, we chose one neighboring square before choosing one from the possible reactions in order to restrict the kind of complex formation reactions the molecule in the first chosen square could go through. Such a reversed procedure was not done for other second-order reactions such as the replication reaction and diffusion because their rate constant, κ and D, was always identical.

Cellular Potts Model

Cellular Potts Model (CPM) is two-scale stochastic CA, in which the rules of updating the CA pertain to the scale of neighboring grid squares and to the scale of a "vesicle" that consists of a number of grid squares [13,14]. Each vesicle has a unique state, which is assigned to the grid squares that constitute the vesicle. There is also one state representing non-vesicle state, called medium state. The CA is updated with stochastic algorithm minimizing "energy" H defined as

$$H = \sum_{(i,j)\in S} J_{(i,j)} + \sum_{k} \lambda (v_k - V_k)^2, \tag{1}$$

where (i, j) denotes a pair of squares that are a neighbor of each others, where eight nearest squares are considered as neighbor (Moore neighbors)[1]; S is a set of all such pairs (not permutations, but combinations); $J_{(i,j)}$ is energy associated with an interface between different vesicles or between a vesicle and the medium, and it depends on the state of square i and j as explained soon; k denotes a vesicle, and it runs through all vesicles in the summation; v_k denotes the volume of a vesicle, i.e. the number of squares that constitute the vesicle; V_k denotes the so-called target volume (neither v nor V exists for the medium); λ is a parameter and is set to 1 throughout this study. The current study defines $J_{(i,j)}$ as follows: $J_{(i,j)} = 3$ if $i \neq j$, and i and j are vesicle states (i.e. if the two squares belong to different vesicles); $J_{(i,j)} = 1$ if $i \neq j$, and either i or j is the medium state; $J_{(i,j)} = 0$ if $i = j$. For these values of

[1]This definition of neighborhood is rather arbitrary. For example, the neighborhood defined in [15] might be more realistic since the Moore neighborhood introduces anisotropy. However, this would hardly matter for the current study.

J, a vesicle tends to be more often in contact with media than with other vesicles. The actual algorithm of updating the CA runs as follows:

1. Chose one square (denoted by x_1) from the grid in a random order such that the same square is not chosen twice until every square is chosen.

2. Randomly chose one (denoted by x_2) of the four nearest squares of x_1 (von Neumann neighbors).

3. Calculate the difference (denoted by ΔH) by subtracting the current value of H from the value of H if the state of x_2 were copied to x_1.

4. If $\Delta H < 0$, copy the state of x_2 to x_1. If $\Delta H > 0$, copy the state of x_2 to x_1 with a probability $\exp(-\Delta H/T)$, where T is a parameter and is set to 1 throughout this study.

The application of this algorithm for N^2 times, where N^2 is the number of squares in the grid, is defined as one time-step of the CPM. The dynamics of the CPM is run for one time-step per every $\mathrm{Int}(M/\alpha)$ time-steps of the replicator dynamics, where $\mathrm{Int}(x)$ is the nearest integer to x; α is defined in the previous section; M was tuned to obtain a reasonable model behavior and was set to 6.62 throughout the current study.

The division of vesicles are as described in the main text, and it can happen once per CPM time-step for every vesicle.

The target volume V decays by an amount drawn from the Binomial distribution with the number of trials V and the success probability d_V, and this happens to every vesicle once every time-step of the replicator CA (hence, d_V is called the decay rate of target volume).

Behavior of the Surface Model for Greater Diffusion Rates

The appearance of spatial pattern differs between $D = 0.1$ and $D = 1$ as shown in Fig. S3 (which is found in this document). The pattern is clearly traveling waves for $D = 0.1$, whereas it appears like spots for $D = 1$. However, as Movie S5 shows, these spots actually move in a random, yet persistent direction. This suggests that the spot-like pattern can be considered as short-lived traveling wave. This difference in the appearance of spatial pattern has an interesting consequence, in that the dependency of the system's stability (i.e. the survival range of k_L and l and $\mu_l^{\max}$) on the system's size (i.e. N) was smaller for $D = 1$ than for $D = 0.1$ (data not shown). Although against the common expectation, this is simply because traveling waves are small for $D = 1$ since they are short-lived.

To understand why the system with a greater diffusion rate displays spot-like short-lived waves, we first investigated the stability of an isolated traveling wave for different diffusion rates—i.e. the death process of an isolated wave. Fig. S4 (which is found in this document) shows the spatio-temporal dynamics of an isolated traveling wave under some idealized initial and boundary condition. The result shows that the tip of a traveling wave rotates in an opposite direction for $D = 0.1$ and 1. For $D = 0.1$ the counterclockwise rotation allows the wave to persist indefinitely if not limited by space. For $D = 1$ the clockwise rotation will, in a long term, annihilate the wave. However, the width of the wave increases as it travels both for $D = 0.1$ and $D = 1$, which is most easily seen at the bottom boundary of the grid. This means that the traveling wave would be stable for both diffusion rates in one-dimensional space. Thus, in two-dimensional space, the stability of a traveling wave, depending on D, hinges on the characteristics of the wave tip. This is because the wave tip is inevitably small in size, so that diffusion can homogenize the spatial structure at the wave tip more easily than at the other parts of the wave. Greater diffusion will bring the local replicator dynamics closer to the deterministic well-mixed system, which implies the eventual extinction is more likely at wave tips. Consequently, an isolated traveling wave is unstable for $D = 1$. However, it must also be noted that a wave can persist for $D = 1$ for quite a long time before annihilation depending on the initial condition as seen from Fig. S4 (cf. Movie S5).

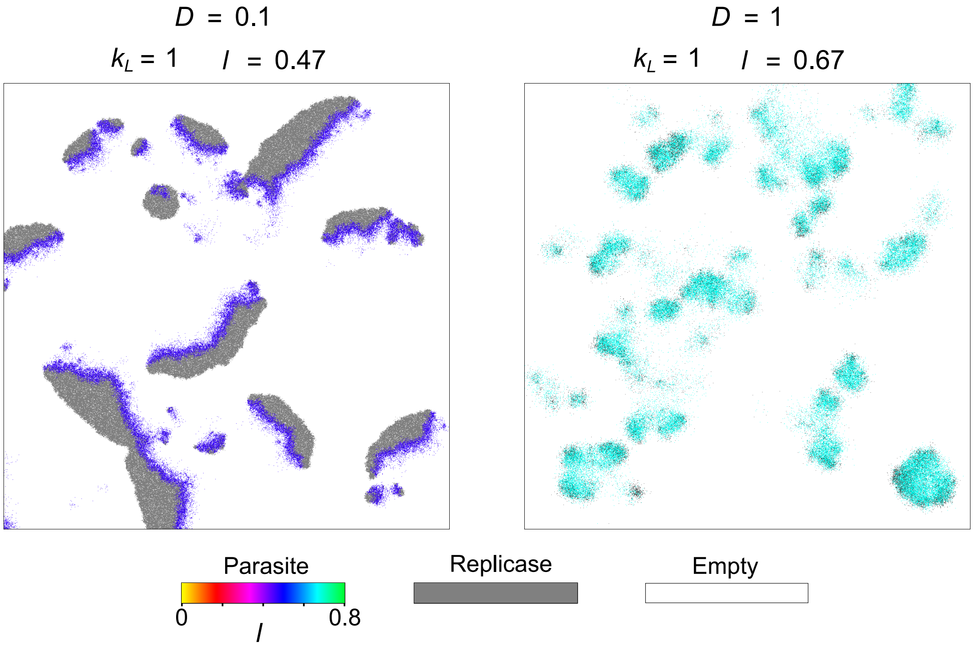

Figure 3. (Figure S3.) Snapshots of the simulations of the surface model for different diffusion rates. The mutation was disabled ($\mu_{k_L} = \mu_l = 0$). $N = 1024$. The other parameters not shown in the picture were the same as in Fig. 2.

The last point naturally leads us to consider the establishment of new waves—i.e. the birth process of waves—for different diffusion rates. We can immediately see that the smallness of a newly generated wave is again important. Given the previous argument concerning wave tips, this implies two points. Firstly, for a greater diffusion rate, the establishment of a new wave and the stability of an isolated (established) wave can be considered as directly connected aspects. Given this point, it can be expected that the system with $D = 1$ displays spot-like short-lived waves. Secondly, the stability of wave dynamics still hinges on the transient growth of a small population of replicase and parasite molecules.

The second point implies that the evolutionary dynamics for greater diffusion rates is expected to be essentially the same as that observed for a smaller diffusion rate. However, we have been unable to observe any long-term evolutionary trend for a greater diffusion rate ($D = 1$). Since the system is less evolutionarily stable for $D = 1$ (Fig. 12A), we had to choose small mutation rates and mutation steps (e.g. $\mu_{k_L} = \mu_l = 0.0005$ and $\delta = 0.02$; the value of N had also to be increased, e.g., to 1536). To check if this was because of too small mutation rates and step, we also examined the system with a smaller diffusion rate ($D = 0.1$) with the same mutation rates and step. The result showed that the system displayed qualitatively the same long-term evolutionary dynamics as presented in the main text. Moreover, the evolutionary trajectory exhibited a step-wise movement (so-called punctuated equilibria), where the size of steps was comparable to the value of δ. This implies that the difference between the "wild type" parasites and the beneficial mutants—which influences the effectiveness of the wave-level selection against random fluctuations—is the limiting factor in the long-term evolution. Given this implication, we next examined whether the wave-level selection could be effective at all for $D = 1$. For this sake, we conducted a competition experiment wherein the two populations of parasites greatly differ in the parameter values (viz., $k_L \approx 0.58$ and $l \approx 0$ *versus* $k_L \approx 0.76$ $l \approx 0.38$). The result showed that the parasite with greater k_L and l value was more adaptive, which is in concordance with the direction of

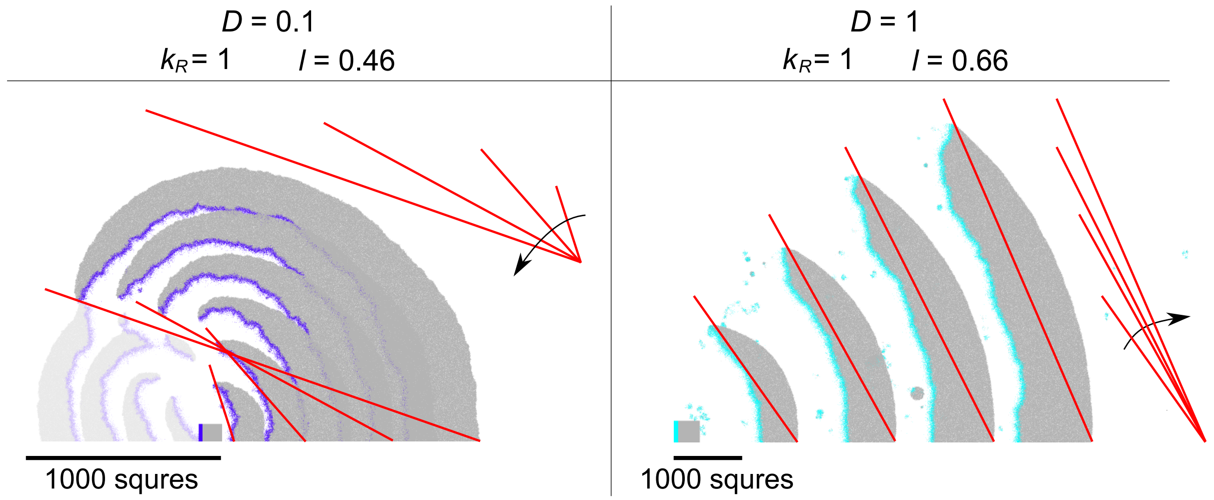

Figure 4. (Figure S4.) Stability of an isolated traveling wave for a greater diffusion rate under an idealized condition. The picture is overlay of simulation snapshots taken consecutively beginning at time zero (for $D = 0.1$, the time span between each picture is 1784; for $D = 1$, it is 4950). Part of the snapshots are blurred for visibility. The red lines and black arrow represents the temporal dynamics of the wave geometry. The parameters not indicated in the figure were the same as in Fig. S3 (the value of l is chosen so as to be slightly below the lower survival boundary depicted by Fig. 11). The initial condition was set in a rectangle domain as depicted by the first snapshot (the size of rectangles scale with $\sqrt{D}$). The boundary condition was modified such that the left and right boundaries are connected (like torus) while the top and bottom boundaries were kept as before (i.e. blocking boundary). For $D = 1$, the simulation was stopped before the system reached equilibrium due to computational overload (the CA size was $N = 5120$); however, a smaller scale simulation (by $1/\sqrt{10}$) showed that an isolated traveling wave does annihilates in a long term (not shown).

the wave-level selection. To conclude, the above considerations imply that the wave-level selection does exist for the greater diffusion rates, but is probably too weak to be effective against random fluctuations for the values of δ for which the system can survive.

Finally, although we have been considering the system with greater diffusion rates at length, we must note that the system with greater diffusion rates shows so little mutational stability (Fig. 12A) that it might be irrelevant to consider this region of the parameter space in the current model anyway.

References

1. Takeuchi N, Hogeweg P (2007) Error-threshold exists in fitness landscapes with lethal mutants. BMC Evol Biol 7: 15.

2. Allee WC (1931) Animal aggregations: a study in general sociology. Chicago: University of Chicago Press.

3. Mitra RD, Church GM (1999) *In situ* localized amplification and contact replication of many individual DNA molecules. Nucleic Acids Res 27: e34.

4. Samatov TR, Chetverina HV, Chetverin AB (2005) Expressible molecular colonies. Nucleic Acids Res 33: e145.

5. Brahmasandra SN, Burke DT, Mastrangelo CH, Burns MA (2001) Mobility, diffusion and dispersion of single-stranded DNA in sequencing gels. Electrophoresis 22: 1046-1062.

6. Hosoda K, Matsuura T, Kita H, Ichihashi N, Tsukada K, et al. (2007) Kinetic analysis of the entire RNA amplification process by Qβ replicase. J Biol Chem 282: 15516-15527.

7. Whitney SE, Sudhir A, Nelson RM, Viljoen HJ (2004) Principles of rapid polymerase chain reactions: mathematical modeling and experimental verification. Comput Biol Chem 28: 195-209.

8. McCaskill JS, Füchslin RM, Altmeyer S (2001) The stochastic evolution of catalysts in spatially resolved molecular systems. Biol Chem 382: 1343-1363.

9. Wilson DS (1975) A theory of group selection. Proc Nat Acad Sci USA 72: 143-146.

10. Boer RJ de, Pagie L (2005). GRIND Ver 2.05. URL `http://theory.bio.uu.nl/rdb/software.html`.

11. Kuznetsov YA (1999). CONTENT Ver 1.5. URL `http://www.math.uu.nl/people/kuznet/CONTENT/`.

12. Takeuchi N, Hogeweg P (2007) The role of complex formation and deleterious mutations for the stability of RNA-like replicator systems. J Mol Evol 65: 668-686.

13. Graner F, Glazier JA (1992) Simulation of biological cell sorting using a two-dimensional extended potts model. Phys Rev Lett 69: 2013-2016.

14. Anderson ARA, Cahplain MAJ, Rejniak KA, editors (2007) Single-Cell-Based Models in Biology and Medicine, Basel: Birkhäuser Verlag, chapter II. pp. 77-167.

15. Markus M, Hess B (1990) Isotropic cellular automaton for modelling excitable media. Nature 347: 56–58.
